# Swiss Vascular Biobank: Evaluation of Optimal Extraction Method and Admission Solution for Preserving RNA from  Human Vascular Tissue

**DOI:** 10.3390/jcm12155109

**Published:** 2023-08-03

**Authors:** Jaroslav Pelisek, Yankey Yundung, Benedikt Reutersberg, Lorenz Meuli, Fabian Rössler, Laetitia Rabin, Reinhard Kopp, Alexander Zimmermann

**Affiliations:** 1 Department of Vascular Surgery, University Hospital Zurich, 8091 Zurich, Switzerland; yankey.yundung@usz.ch (Y.Y.); laetitia.rabin@usz.ch (L.R.); reinhard.kopp@usz.ch (R.K.); alexander.zimmermann@usz.ch (A.Z.); 2 Department of Surgery and Transplantation, University Hospital Zurich, 8091 Zurich, Switzerland

**Keywords:** biobanking, Swiss Vascular Biobank, vascular tissue, cardiovascular diseases, atherosclerosis, RNA integrity number (RIN), DV200 index, cryoprotective solution, RNAlater

## Abstract

Proper biobanking is essential for obtaining reliable data, particularly for next-generation sequencing approaches. Diseased vascular tissues, having extended atherosclerotic pathologies, represent a particular challenge due to low RNA quality. In order to address this issue, we isolated RNA from vascular samples collected in our Swiss Vascular Biobank (SVB); these included abdominal aortic aneurysm (AAA), peripheral arterial disease (PAD), healthy aorta (HA), and muscle samples. We used different methods, investigated various admission solutions, determined RNA integrity numbers (RINs), and performed expression analyses of housekeeping genes (*ACTB, GAPDH*), ribosomal genes (*18S*, *28S*), and long non-coding RNAs (*MALAT1*, *H19*). Our results show that RINs from diseased vascular tissue are low (2–4). If the isolation of primary cells is intended, as in our SVB, a cryoprotective solution is a better option for tissue preservation than RNAlater. Because RNA degradation proceeds randomly, controls with similar RINs are recommended. Otherwise, the data might convey differences in RNA degradation rather than the expressions of the corresponding genes. Moreover, since the 18S and 28S genes in the diseased vascular samples were degraded and corresponded with the low RINs, we believe that DV200, which represents the total RNA’s disintegration state, is a better decision-making aid in choosing samples for omics analyses.

## 1. Introduction

Human tissue biobanks constitute an important source for clinical and translational research which seeks to understand the pathophysiological mechanisms behind particular diseases in order to be able to discover suitable biomarkers and optimal therapeutic strategies [1,2]. The challenge for every biobank is ensuring that the collected tissues are of the best possible quality, particularly for various omics analyses. Unfortunately, no universal method for processing the collected biomaterial has yet been established. Furthermore, the optimal treatment and processing of the specimens may vary depending on the particular organ tissue to be preserved.

Cardiovascular disorders (CVDs) are the leading cause of death worldwide [3]. In the past few decades, endovascular treatment has become an established, minimally invasive surgical intervention for CVDs [4]. Nowadays, most patients with AAA (~80%) receive endovascular aortic repair (EVAR). Carotid artery stenting and endovascular procedure in PAD patients have also become viable alternatives to open surgical intervention. Therefore, collecting human vascular biomaterial for further analyses following surgical interventions has become an important means of improving our understanding of the underlying processes which lead to the formation of atherosclerotic lesions and aortic aneurysms, as well as the rupture-prone phenotype [5,6,7,8,9]. Due to atherosclerotic changes and other pathologies within the arterial wall, the quality of the diseased vascular tissue can be diminished and broadly heterogeneous [10,11,12]. The extraction of high-quality RNA for transcriptome analysis and single-cell RNA sequencing made particularly difficult by to the accumulation of dead and apoptotic cells, oxidative stress, inflammation, connective tissue degradation, and calcification [11,12]. Nonetheless, to acquire reliable data from omics analyses of diseased vascular tissue samples, RNA quality should be as high as possible. Consequently, working with tissue samples obtained from patients with CVDs constitutes a particular challenge.

The most frequently used method to assess the quality of RNA currently acknowledged is the determination of the RIN (RNA integrity number). Typically, an RIN score of 6–7 or higher is recommended for sequencing analyses to achieve reliable data [10,13]. However, depending on the extent of tissue degradation, inflammation, oxidative stress, and other factors associated with CVDs, the RINs of atherosclerotic and aneurysmal samples are, in most cases, significantly lower. Therefore, carefully performed RNA extraction is necessary to bring out the best in the collected diseased vascular specimens. In addition, because RINs are already low in atherosclerotic tissue, other methods should be considered for the determination of the suitability of the samples for transcriptomics and other sensitive analyses. Here, for instance, the DV200 index, another way of describing the extent of RNA degradation by evaluating the percentage of fragments of >200 nucleotides, is also a helpful quality assessment standard [14]. Furthermore, the messenger RNAs (mRNAs) of different genes exhibit different cellular stability, expression patterns, and dependency upon RNA degradation [15].

In order to address these important issues, we selected various vascular tissue samples from our Swiss Vascular Biobank (SVB), isolated the RNA using different methods, determined the RNA quality, and performed real-time polymerase chain reaction (RT-PCR) analyses involving various genes (housekeeping and ribosomal genes, long non-coding RNAs (lncRNAs)). In addition, we compared the effects of different preserving solutions on the RNA integrity, incubating the tissue at 4 °C or room temperature (RT) for up to 7 days prior to extracting the RNA.

## 2. Materials and Methods

### 2.1. Tissue Samples

Vascular tissue samples were obtained from patients who underwent open surgical interventions in our Department of Vascular Surgery (USZ/UZH). Samples are continuously collected in our Swiss Vascular Biobank (SVB). The following tissues were included in our study: infrarenal abdominal aortic aneurysm tissue (AAA, *n* = 50), tissue affected by peripheral arterial disease (PAD, *n* = 40), muscle tissue from the sartorius muscle obtained from PAD patients following open surgical repair (Mu, 100 different pieces from 5 individuals), and non-aneurysmal healthy infrarenal aortic samples (HA, *n* = 20) obtained during kidney transplantation and provided by the Department of Visceral Surgery (USZ/UZH). All tissue samples were stored at −80 °C within five minutes of excision and kept frozen until the RNA extraction. All patients whose vascular tissue samples were collected in the SVB gave appropriate written informed consent. In emergency cases, the tissue samples were collected temporarily and the written consent was obtained after the surgical intervention. In cases of rejection, the tissue was disposed of. The local ethics committee (Cantonal Ethics Committee Zurich, Switzerland; BASEC-Nr. 2020-00378) approved the tissue sample collection and analysis procedure. In addition, as a positive control for RNA quality, human endothelial cells (EA.hy926; ATCC, Manassas, VA, USA) were used.

### 2.2. RNA Extraction

**Figure 1:**
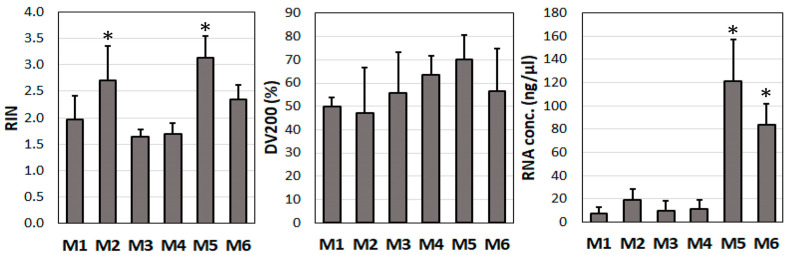
Comparison of methods for optimal RNA extraction from human vascular tissue samples (*n* = 5). M1: Homogeniser and Rneasy Kit, 10 min digestion with proteinase K; M2: Homogeniser and Rneasy Kit, 90 min digestion with proteinase K; M3: Homogenisation using a mortar and liquid nitrogen, Rneasy Kit, 10 min digestion with proteinase K; M4: Homogenisation using a mortar and liquid nitrogen, Rneasy Kit, 90 min digestion with proteinase K; M5: Homogeniser and TRIZOL; M6: Homogenisation using a mortar and liquid nitrogen, TRIZOL. RIN: RNA integrity number; DV200 index: % of RNA fragments > 200 bp. * *p* < 0.05 (compared with standard method M1). The statistical significance of each individual sample was compared against the standard method M1 using an independent *t*-test.

In order to determine the optimal extraction protocol for achieving the maximal quantity and quality of RNA from the study tissue samples, various methods and assays were applied (see Figure 1). The deployment of homogeniser (POLYTRON PT2500E; Faust, Schaffhausen, Switzerland) was compared with manual homogenisation using a mortar and liquid nitrogen. Different periods of tissue digestion via proteinase K were used to test for improvements in the release of RNA, and TRIZOL reagent (Thermo Fisher Scientific, Zug, Switzerland) was compared with the Rneasy Kit from Qiagen (Hombrechtikon, Switzerland). Using the TRIZOL reagent, the tissue samples were homogenised in a volume of 1 mL, mixed with 200 µL chloroform, and centrifuged for 15 min at 12,000× *g* and 4 °C. The upper (clear) phase containing the RNA was carefully transferred into a new tube, 500 µL isopropanol was added, and the contents were mixed by inverting the tube several times to precipitate the RNA. Following incubation on ice and further centrifugation, the supernatant was discarded and the RNA was resuspended in 75% ethanol to improve RNA solubility. It was then centrifuged and the supernatant was again discarded. The RNA pellet was air-dried, resuspended in 50 µL RNase-free water, and stored at −80 °C until further use. The RNA concentration was determined using a NanoDrop Lite Plus Spectrophotometer (Witec, Sursee, Switzerland). Because the TRIZOL solution and a motorised homogeniser provided the highest RNA concentration and quality (Figure 1), all further experiments described below were performed using this method of RNA extraction.

### 2.3. Determination of the RNA Quality

In order to evaluate the RNA quality and the degree of degradation of our tissue samples, TapeStation 4150 (Agilent, Basel, Switzerland) was used. The RNA integrity numbers (RINs) and DV200 index values (percentage of fragments of >200 nucleotides) of each specimen were determined using a standard Agilent RNA ScreenTape Assay or Agilent High Sensitivity RNA ScreenTape Assay, depending on the RNA concentration and in accordance with the manufacturer’s protocols. All measurements were performed in duplicates, and the experiments were repeated in cases of high variability or discrepancy.

### 2.4. Time-Dependent Comparison of Different Protective Solutions

In order to compare the stability and quality of the RNA under different conditions, three protective solutions were used: (1) an optimised cryoprotective solution (CpS) composed of cell culture medium (DMEM/RPMI; Thermo Fisher Scientific, Reinach, Switzerland), 1.8 M dimethyl sulfoxide (DMSO), 0.1 M glucose (Merck/Sigma Aldrich, Buchs, Switzerland), 20 mM 2-(4-(2-Hydroxyethyl)-1-piperazinyl)-ethane sulfonic acid (HEPES), and, optionally, 1% fetal bovine serum (FBS) (Thermo Fisher Scientific) [16,17,18,19]; (2) Phosphate buffered saline (PBS); (3) RNAlater (25 mM sodium citrate, 10 mM ethylenediaminetetraacetic acid (EDTA), 5.3 M ammonium sulphate, pH 5.2). Three representative specimens from each study tissue sample (Mu, HA, AAA, PAD) were incubated in triplicates in the different storage solutions for 8 h, 1, 2, 3, and 7 days. At each time point, the RNA was extracted as described above. All tissue samples used for analysis were incubated in triplicates and in sufficient quantities to ensure three samples for each individual time point in the corresponding admission solution. At the selected time points (8 h, 1, 2, 3, and 7 days) three samples were removed for analysis. The results of each protective solution were then compered with those for time point 0.

### 2.5. Quantitative Real-Time PCR Analysis

In order to identify the interrelationships between RNA quality, the various protective solutions, and gene expression at the mRNA level, quantitative real-time PCR (qRT-PCR) analyses were performed. First, cDNA was synthesised using 1 µg RNA and a RevertAid First Strand cDNA Synthesis Kit (VWR International, Dietikon, Switzerland). Random Hexamer primers were used in order to encompass non-coding RNAs and degraded RNA fragments independent of the state of the poly(A) tail.

PowerUp SYBR Green Master Mix (Thermo Fisher Scientific) was used to determine the expression of the housekeeping genes Beta-actin (*ACTB*) and Glycerinaldehyd-3-phosphat-Dehydrogenase (*GAPDH*). Furthermore, to evaluate the stability of the ribosomal genes *18S* and *28S* over time, which is used to calculate the RIN (Agilent), the expression of these genes was also analysed. All primers for the SYBR Green expression analysis were purchased from Qiagen. In addition, the expression of two long non-coding RNAs (lncRNAs), *MALAT1* and *H19*, which play an important role in vascular disorders [7,20,21], was determined using TaqMan Fast Advanced Master Mix and a corresponding primer (Thermo Fisher Scientific). The PCR reaction was performed using QuantStudio QS5 (Fisher Scientific) and the standard PCR conditions recommended by the manufacturer.

The following primers were used:

*ACTB*: Hs_*ACTB*_2_SG, amplicon length 104 bp
*GAPDH*: Hs_*GAPDH*_vb.1_SG, amplicon length 112 bp (exons 2/3)
*GAPDH*: Hs_*GAPDH*_1_SG, amplicon length 95 bp (exons 6/7)
ribosomal Gene *18S*: Hs_RPS18_1_SG, amplicon length 61 bp
ribosomal Gene *28S*: Hs_RPS28_1_SG, amplicon length 77 bp
*MALAT1*: Hs00273907_s1, amplicon length 117 bp
*H19*: Hs00399294_g1, amplicon length 62 bp (exons 2/3)

### 2.6. Statistical Analysis

**Figure 2:**
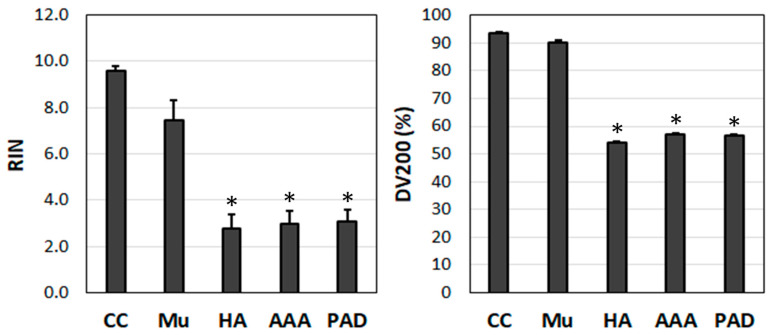
Determination of RINs and DV200 index values in the study tissue samples. CC: human endothelial cells (EA.hy926, *n* = 6); Mu: human muscle (*n* = 20) ^#^; HA: healthy aorta (*n* = 20); AAA: abdominal aortic aneurysm (*n* = 50); PAD: peripheral arterial disease (*n* = 40). RIN: RNA integrity number; DV200 index: % of RNA fragments > 200 bp. * *p* < 0.05, ^#^ 20 pieces from 5 patients. The statistical significance of each individual sample was compared against the CC using an independent *t*-test.

**Figure 3:**
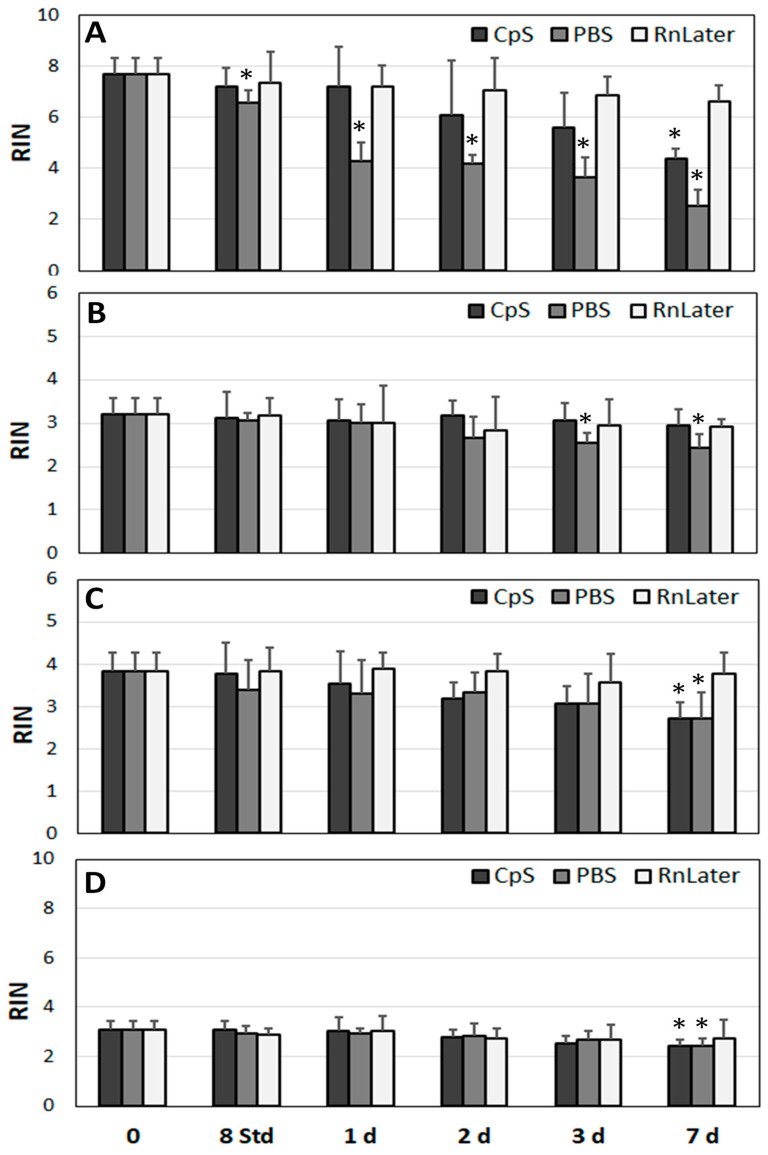
Determination of RINs in the study tissue samples over time (incubated at 4 °C for up to 7 days). (**A**) Mu, (**B**) HA, (**C**) AAA, (**D**) PAD. RIN: RNA integrity number (*n* = 9, three tissue samples in triplicates for each time point); * *p* < 0.05 (related to time point 0 for each admission solution); CpS: cryoprotective solution; PBS: phosphate buffered saline; RnLater: RNAlater solution.

**Figure 4:**
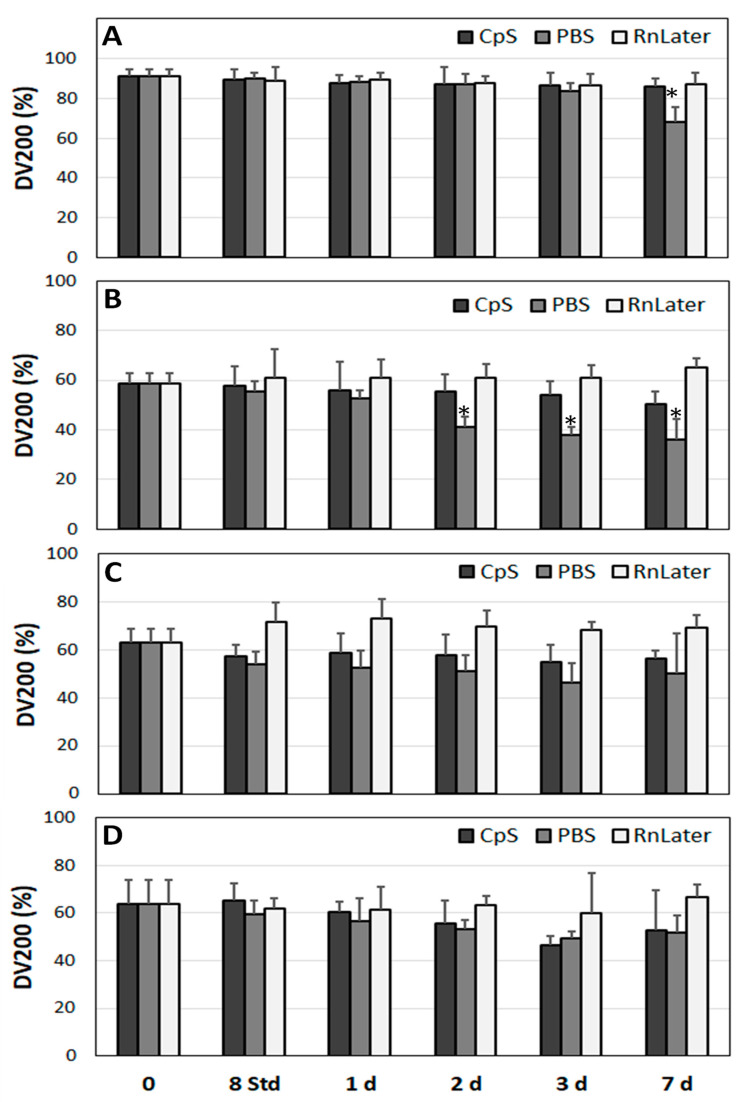
Determination of DV200 index values in the study tissue samples over time (incubated at 4 °C for up to 7 days) (**A**) Mu, (**B**) HA, (**C**) AAA, (**D**) PAD. *n* = 9, three tissue samples in triplicates for each time point. DV200 index: % of RNA fragments > 200 bp. CpS: cryoprotective solution; PBS: phosphate buffered saline; RnLater: RNAlater solution. * *p* < 0.05 (related to time point 0 for each admission solution).

**Figure 5:**
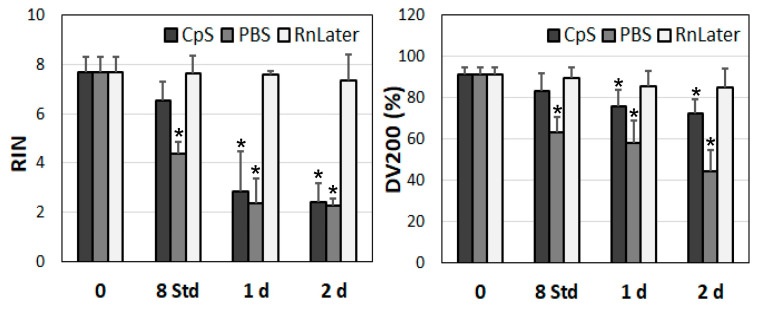
Determination of RNA integrity numbers (RINs) and DV200 index values over time (up to 7 days) (*n* = 9, three tissue samples in triplicates for each time point). Tissue samples were kept at room temperature using different preservation solutions and non-diseased muscle tissues. CpS: cryoprotective solution; PBS: phosphate buffered saline; RnLater: RNAlater solution. * *p* < 0.05 (related to time point 0 for each admission solution).

**Figure 6:**
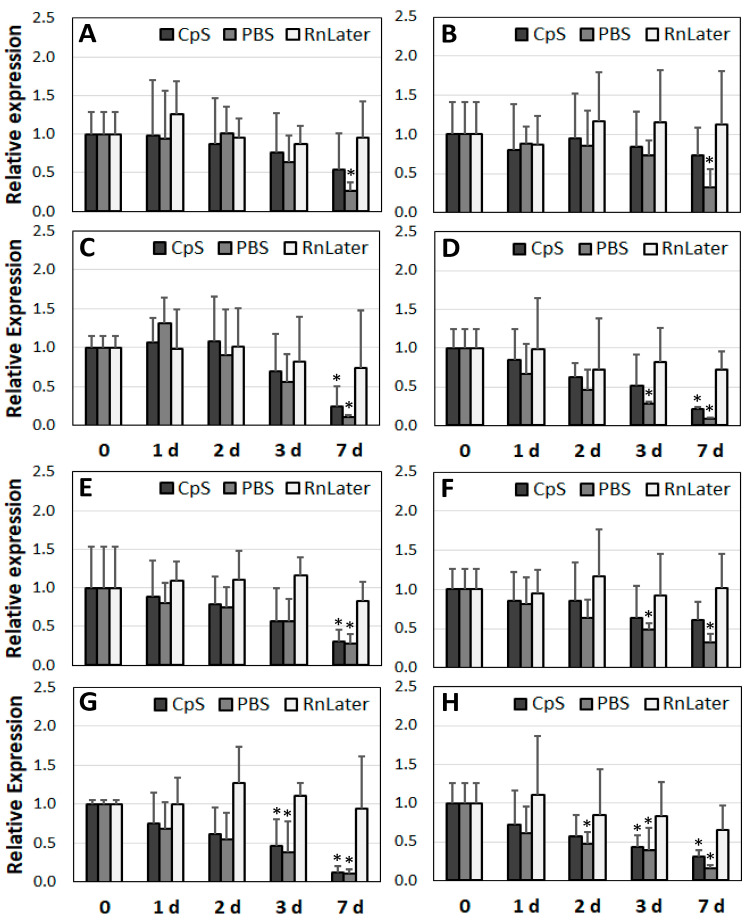
Expression analysis of the housekeeping genes *ACTB* (**A**–**D**) and *GAPDH* (**E**–**H**) using different preservation solutions (*n* = 3). (**A**,**E**): muscle tissue; (**B**,**F**): healthy aortic tissue; (**C**,**G**): abdominal aortic aneurysm tissue; (**D**,**H**): peripheral arterial disease tissue. CpS: cryoprotective solution; PBS: phosphate buffered saline; RnLater: RNAlater solution. * *p* < 0.05 (related to time point 0 for each admission solution).

**Figure 7:**
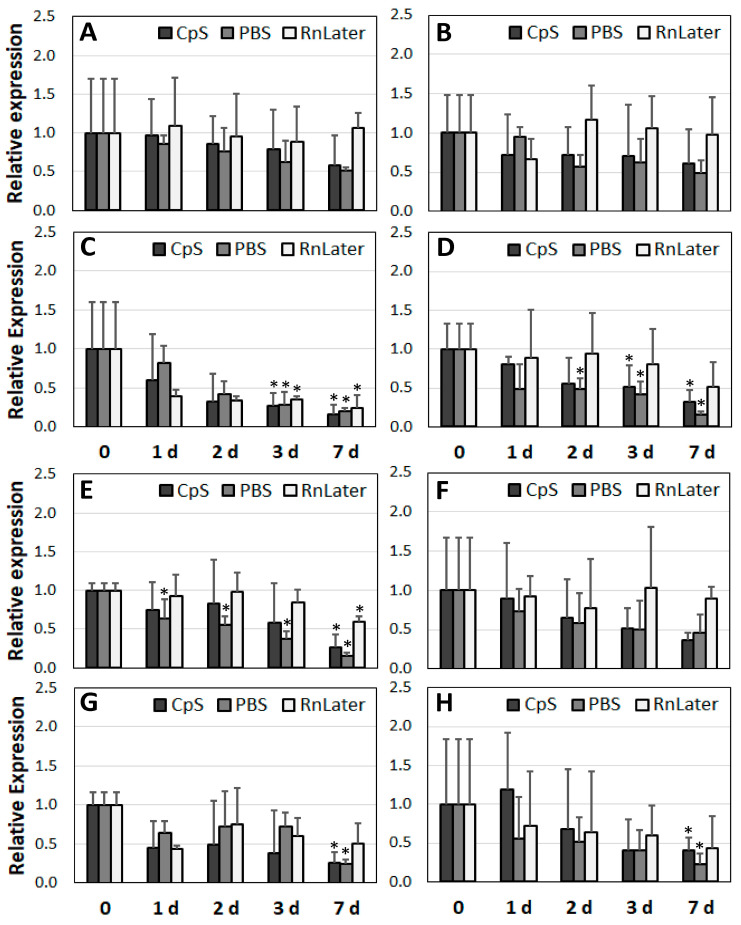
Expression analysis of the ribosomal genes *18S* (**A**–**D**) and *28S* (**E**–**H**) using different preservation solutions (*n* = 3). (**A**,**E**): muscle tissue; (**B**,**F**): healthy aortic tissue; (**C**,**G**): abdominal aortic aneurysm tissue; (**D**,**H**): peripheral arterial disease tissue. CpS: cryoprotective solution, PBS: phosphate buffered saline; RnLater: RNAlater solution. * *p* < 0.05 (related to time point 0 for each admission solution).

**Figure 8:**
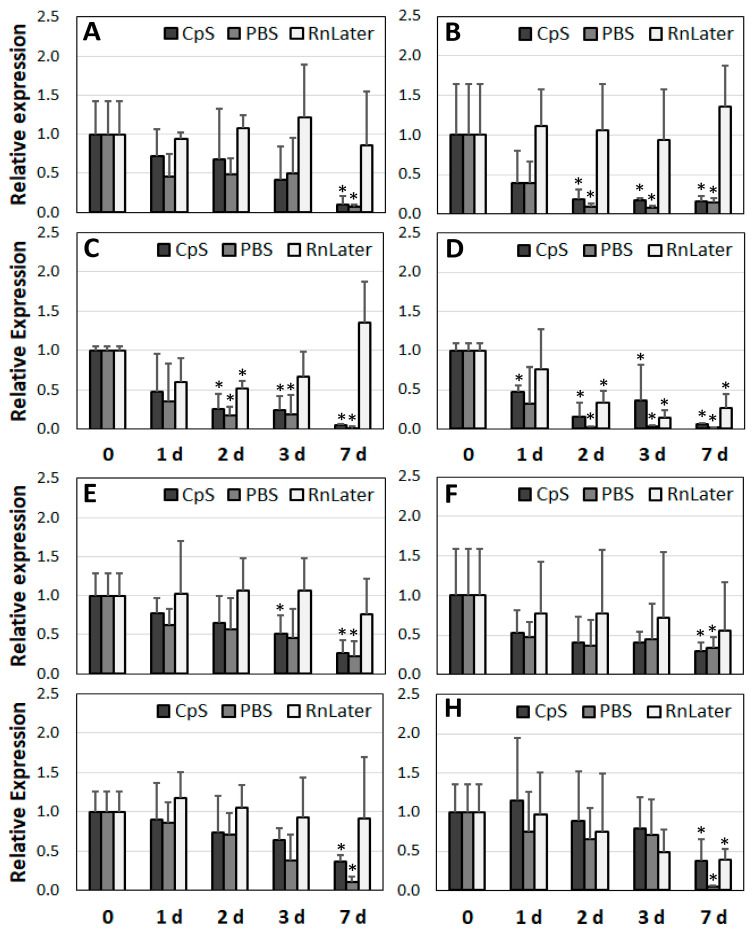
Expression analysis of two selected long non-coding RNAs, *MALAT1* (**A**–**D**) and *H19* (**E**–**H**), using different preservation solutions (*n* = 3). (**A**,**E**): Mu; (**B**,**F**): HA; (**C**,**G**): AAA; (**D**,**H**): PAD. CpS: cryoprotective solution; PBS: phosphate buffered saline; RnLater: RNAlater solution. * *p* < 0.05 (related to time point 0 for each admission solution).

All statistical analyses were performed using IBM’s SPSS software version 29 (SPSS Inc., Chicago, IL, USA). In Figure 1 and Figure 2, the individual samples were compared against a standard sample (M1 in Figure 1 and endothelial cells in Figure 2) using an independent *t*-test and a Levene-test of equality of variances. Regarding the analysis of the RNA quality and expression at the mRNA level over time (Figure 3, Figure 4, Figure 5, Figure 6, Figure 7 and Figure 8), these were compared separately for each admission solution, and all time points were compared against time point 0. Again, an independent *t*-test and a Levene test of equality of variances was used. All statistical analyses were two-sided and used *p* < 0.05 as the significance level. Pearson correlation coefficients were determined to evaluate statistical dependences between variables. For the correlation analyses, *p*-values of * 0.05, ** 0.01, and *** 0.001 were applied.

## 3. Results

### 3.1. Evaluation of Optimal Method of Extracting RNA from Vascular Tissue

First, we compared different homogenisation tools (homogeniser vs. mortar) and isolation methods (Rneasy Kit vs. TRIZOL) for their suitability to obtain RNA of the highest quality and quantity from our vascular tissue samples (methods M1–M6, Figure 1).

The highest RIN values were obtained using homogeniser and either TRIZOL or the RNease Kit (3.1 ± 0.4 or 2.7 ± 0.6) (Figure 1, upper panel: M2 and M5). Furthermore, regardless of the homogenisation method used, TRIZOL consistently outperformed the Rneasy Kit (Figure 1). When using homogeniser and the RNease Kit, a period of 90 min for the digestion of the tissue with proteinase K led to higher RIN values (2.7 ± 0.6) than a digestion time of 10 min (2.0 ± 0.5) (*p* = 0.020). In contrast, when using mortar for homogenisation, extending the proteinase K incubation time did not improve the RNA quality (1.7 ± 0.1 vs. 1.7 ± 0.2, *p* = 0.780).

Overall, the DV200 index values ranged between 40% and 80%, and there were no significant differences between the individual methods used (Figure 1, middle panel). Nevertheless, when the RNA was isolated with motorised homogeniser and TRIZOL, the DV200 values were higher (70.1 ± 10.4%) than those obtained using the mortar (56.5 ± 18.1%), though the difference was not statistically significant. The changes in proteinase K incubation time in the RNease Kit did not significantly affect the DV200 index values.

Regarding RNA concentration, the highest values were obtained when using homogeniser and TRIZOL (121.5 ± 35.7 ng/µL); these were higher than the values obtained using the Rneasy Kit (19.4 ± 9.1 ng/µL) (Figure 1, lower panel). When using the mortar, the highest RNA concentration values were observed when using TRIZOL (83.5 ± 18.4 ng/µL); these were higher than the values obtained using the Rneasy Kit (7.6 ± 5.1 ng/µL).

In summary, the TRIZOL solution and the motorised homogeniser provided the highest RNA concentration and quality.

### 3.2. Determination of RIN and DV200 Values in the Study Tissue Samples

After establishing the optimal isolation protocol, the RNA from all the tissue samples included in our study was isolated using TRIZOL and homogeniser. In order to gain an overview of the RNA quality, we determined the RIN score and DV200 index values in the different diseased and healthy tissue samples (muscle, HA, AAA, and PAD) as well as in the human endothelial cell line (EA.hy926) (Figure 2).

The RNA from the endothelial cell and the muscle samples had the highest RIN and DV200 values. They differed significantly from the healthy aorta (HA) and diseased vascular tissue samples (AAA, PAD) (Figure 2). The endothelial cell samples had the highest RIN values (9.6 ± 0.2), followed by the muscle samples (7.5 ± 0.9). The RINs of the other study tissue samples ranged between 2 and 4 (HA: 2.8 ± 0.6, *n* = 20; AAA: 3.0 ± 0.5, *n* = 50; PAD 3.1 ± 0.5, *n* = 40). A similar pattern was observed in the DV200 index values. The endothelial cell samples had the highest DV200 values (93.6 ± 2.1%), followed by the muscle samples (90.2 ± 3.8%). The DV200 values of the diseased vascular tissue samples ranged between 36 and 76 (HA: 54.0 ± 10.4%; AAA: 56.9 ± 12.1%; PAD: 56.6 ± 14.3%).

Taken together, in contrast to the healthy muscle tissue, which had RINs of 7.5 ± 0.9 and DV200 index values of 90.2 ± 3.8%, the quality of the diseased vascular tissue samples was significantly reduced, with RINs of 3.0 ± 0.5 and DV200 index values of 56.8 ± 13.2%.

### 3.3. Stability of RNA over Time

In our experimental approach, outlined below, the extent of RNA degradation in the study samples (Mu, HA, AAA, PAD)—all of which were incubated at 4 °C—was analysed in different protective solutions (CpS, PBS, RNAlater) at different time points (Figure 3, Figure 4 and Figure 5).

The RINs of the muscle samples at time point 0 were 7.7 ± 0.6 (Figure 3A). The best preserving medium appeared to be RNAlater, for which no significant changes were observed over time (up to 7 days). The second-best admission solution was the CpS, for which substantial but not significant changes were observed over time (up to 3 days). In contrast, when the muscle tissue samples were incubated in PBS, the RINs dropped significantly after just one day (4.3 ± 0.7, *p* = 0.001).

In contrast to the muscle tissue samples, the RINs of the HA samples (3.2 ± 0.4) were significantly lower at time point 0 (Figure 3B). The RIN values remained stable in the RNAlater solution and the CpS even after 7 days (RNAlater: 2.9 ± 0.2; CpS: 3.0 ± 0.4). In the PBS solution, the RINs did not change for up to 2 days, and they dropped significantly (compared with time point 0) to 2.4 ± 0.3 after 7 days. Similar results were observed for the RNA isolated from the AAA and PAD samples (Figure 3C,D). The RIN values at time point 0 were 3.1 ± 0.4 and 3.8 ± 0.5, respectively. In the RNAlater solution and the CpS, the RNA was stable for up to 7 days. Regarding PBS, the RINs were unchanged for up to 2 days, with a slight but significant reduction in RIN at day 3 (2.5 ± 0.3, *p* = 0.037).

The DV200 index values appeared much less sensitive to the changes in RNA quality over time. No significant alternations were observed in the muscle tissue samples in the RNAlater solution or the CpS for up to 7 days. Only in PBS did the DV200 values decreased from 92.2 ± 3.2% to 68.0 ± 7.8% at day 7 (*p* < 0.001). For the HA samples, the DV200 index values started at 58.4 ± 4.3% at time point 0 and remained stable in the RNAlater solution and the CpS for up to 7 days. In PBS, the DV200 values decreased slightly but continuously, with significant differences after 2, 3, and 7 days (respectively, from 55.6 ± 3.7 to 41.3 ± 3.9%, 37.8 ± 3.4%, and 36.0 ± 8.6%, *p* = 0.004, 0.003, and 0.008) (Figure 4B). RNA isolated from the AAA and PAD samples showed similar stability over time, with no significant changes for up to 7 days in any of the admission solutions (Figure 4C,D).

In addition, we also tested the stability of RNA over time at room temperature (Figure 5). In this experimental approach, we tested only the muscle tissue samples and used time periods of up to 2 days. No significant changes in the RIN scores or DV200 index values were observed in the RNAlater solution (Figure 5, left panel). In contrast, at room temperature, the RNA quality dropped rapidly in the CpS and PBS. In the cryoprotective solution, the RIN was stable for up to 8 h and significantly diminished after one day (from 7.7 ± 0.6 to 2.8 ± 1.6, *p* < 0.001). These changes were even more conspicuous in the PBS solution, where the RIN values significantly dropped after just 8 h to 4.63 ± 0.46 (*p* < 0.001) and were further reduced to 2.4 ± 1.0 (*p* < 0.001) after one day. Even the DV200 values were significantly more affected at room temperature compared with 4 °C (Figure 5, right panel), though not as much as the RIN values. In the RNAlater solution, no significant changes were observed in the RINs. In the CpS, significant differences were observed after one day. However, these changes were marginal (from 91.2 ± 3.3% to 75.8 ± 8.5%, *p* = 0.014). Again, the most substantial drop was observed for the PBS solution: significant changes after just 8 h and a further reduction in the DV200 index values to 44.2 ± 9.0% (*p* = 0.007) after 2 days (Figure 5, right panel).

Taken together, the RNAlater solution showed the highest stabilising properties against RNA degradation, followed by the CpS. The PBS solution was the least protective. In vascular tissue with already partially degraded RNA, the RIN values were less susceptible to changes over time than in the non-diseased muscle tissue. Furthermore, the DV200 index values, indicating the general extent of RNA disintegration in comparison with the RIN values, indicating only the degradation of ribosomal mRNAs, showed higher robustness regarding the susceptibility of the RNA to changes over time. In addition, incubation at room temperature significantly accelerated the RNA degradation compared with storage at 4 °C.

### 3.4. Changes in Gene Expression over Time

After determining the RNA quality over time, we analysed corresponding changes in expression at the mRNA level. For this purpose, we selected three groups of genes: housekeeping (*GAPDH, ACTB*), ribosomal (*18S, 28S*), and lncRNAs (*MALAT1, H19*) (Figure 6, Figure 7 and Figure 8).

First, regarding the RNAlater admission solution, no significant changes in expression were observed over time for the housekeeping genes, regardless of the sample study group used (Figure 6). The ribosomal genes were less stable, particularly *28S*. In the muscle and HA tissue samples, the expression of the *18S* gene was stable for up to 7 days. In the AAA and PAD samples, the expression dropped down significantly at days 3 and 7 in the RNAlater solution (respectively, 2.9- and 1.9-fold; *p* = 0.049 and *p* = 0.033) (Figure 7C,D). For the lncRNAs preserved in the RNAlater solution, gene expression did not change significantly for up to 3 days at 4 °C. With the exception of PAD, both of the lncRNAs (*MALAT1* and *H19*) were stable for up to 7 days (Figure 8).

Concerning our cryoprotective solution (CpS), the expression of the housekeeping gene *ACTB* did not significantly change in any of the tested samples for up to 3 days (Figure 6A–D). The RNA for the *GAPDH* gene showed lower stability than that for the *ACTB* gene. For the muscle tissue and HA samples, expression at the mRNA level slightly but significantly diminished after 7 days of incubation at 4 °C (3.2- and 1.6-fold, respectively). In the diseased AAA and PAD samples, significant reductions were observed as early as day 3 (3.2- and 1.6-fold, respectively), reaching up to a 7-fold reduction in expression at day 7 (Figure 6E–H). The mRNA of the ribosomal gene *18S* in the diseased vascular tissue samples was less stable than that of the housekeeping genes (Figure 7C,D). A significant reduction was observed after only 1–2 days (up to 6-fold).

The RNA of the *28S* gene was stable for up to 3 days (Figure 7E–H). In the CpS, the *MALAT1* gene was the least stable. In the HA, AAA, and PAD samples, its expression was diminished after only 1–2 days and dropped by a factor 20 at day 7. The expression of *H19* remained unchanged in the diseased vascular tissue samples for up to 3 days.

The greatest reduction in gene expression was observed in the PBS solution (Figure 6, Figure 7 and Figure 8). Nonetheless, the housekeeping genes did not show any changes for 2–3 days, depending on the tissue samples (Figure 6). The expression of *ACTB* was stable for up to 3 days, and that of *GAPDH* for up to 2 days. The expression of the ribosomal mRNA *18S* in the diseased vascular tissue and PBS samples dropped after just 1–2 days, and for *28S* it first dropped at the day 7. In the muscle tissue samples, the expression of *S18* appeared more stable than that of *28S* (Figure 7A,E). In the PBS, the lncRNA *MALAT1* appeared the least stable in terms of its expression (Figure 8). In contrast to *H18*, whose expression was unchanged for 2–3 days in each of the tissues tested, the expression of *MALAT1* dropped after just 1 day, and up to a 90-fold reduction was observed at day 7.

Thus, regarding gene expression, the most stable preservation solution was RNAlater, followed by the CpS and PBS. The most stable RNA appeared to be *ACTB*, followed by *GAPDH.* The RNA of the ribosomal genes was less stable than that of the housekeeping genes, particularly in the diseased vascular tissue. Regarding the expression of lncRNAs, *MALAT1* in particular was found to be the least stable.

### 3.5. Correlation between RIN, DV200, and Gene Expression

In order to evaluate the relationships between RIN, DV200, and gene expression over time in the different protective solutions, correlation analyses were performed (Table 1).

For healthy muscle tissue samples, which were used as a reference, the quality of the RNA (RIN) diminished over time, independent of the protective solution used. The stability of the RNA was highest in the RNAlater solution and lowest in the PBS (respectively, *r* = −0.467 and *r* = −0.922; *p* < 0.05 and *p* < 0.001). Interestingly, the DV200 index values did not correlate with time or RIN, except in the case of CpS (*r* = −0.601, *p* < 0.05), and this suggests that DV200 is more robust than RIN in determining RNA degradability. Regarding the diseased tissue samples (AAA and PAD), in which the RNA was already partially degraded at time point 0, the RNAlater solution exhibited the best protective properties for RNA, showing no correlation between time, RIN, and DV200. For the other protective solutions, the CpS and PBS, the RIN values significantly diminished over time (Table 1). The DV200 index values were heterogeneous and partially interrelated with the RIN values. However, here too DV200 was more robust over time than RIN. The healthy aorta was a special case. As mentioned above, the RIN and DV200 values were much lower than expected due to the cold ischemia, and they correlated with the results obtained from the diseased tissue samples. Here, only the PBS showed significant correlations between RIN, DV200, and time. The CpS and RNAlater solution showed good protective properties against RNA degradation.

Regarding the analysis of expression over time in the study tissue samples, the RNAlater solution demonstrated the best protective properties. In most cases, no correlations were observed between gene expression, RIN, DV200, and time (Table 1). The lowest protective features were observed in the PBS. Significant relationships were found between the expressions of all study genes at the mRNA level and time. In the muscle tissue and healthy aorta samples, significant correlations were observed between RIN and gene expression for most of the genes analysed. Interestingly, regarding the CpS, which we use for our SVB tissue collections, and which has the advantage of later isolation of primary vascular cells after freezing, different mRNA stability values were observed. In the human muscle tissue samples, the ribosomal genes and lncRNAs significantly correlated with the RIN values (*p* < 0.01). In contrast, the expressions of the housekeeping genes *ACTB* and *GAPDH* were stable over time. Similar results were observed for the healthy aorta samples (Table 1). In the diseased arterial tissue samples (AAA and PAD), the results were very heterogeneous; the least stable seemed to be the lncRNAs. Interestingly, the DV200 index values did not correlate with the level of mRNA expression over time. Only in the diseased vascular tissue samples were the ribosomal genes and lncRNAs partially associated with DV200.

In summary, the correlation analysis showed the superiority of the RNAlater solution in stabilising the RNA over time at 4 °C. The CpS demonstrated high protective properties as well, with the advantage that it preserved the tissues for other analyses. DV200 showed higher robustness regarding RNA degradation compared with RIN, based upon an algorithm calculated from the ribosomal genes RS18 and RS28. The RNA stability significantly varied between the selected genes. The housekeeping genes seemed to be the most stable, and the lncRNAs the least stable.

## 4. Discussion

The proper collection and storage of high-quality biomaterial in biobanks is crucial for the reliable analysis of the acquired tissue samples, and for data reproducibility. Diseased vascular tissue represents a particular challenge due to its broad heterogeneity and low RNA quality, which is caused by various atherosclerotic pathologies. In this work, we compared different methods and solutions for their suitability to preserve RNA and prevent degradation.

Our results demonstrated that TRIZOL and a motorised homogeniser provide the best conditions with the highest RNA concentration and quality. TRIZOL, a guanidinium thiocyanate, possesses high protective qualities that reduce nucleic acid degradation. It is widely used to extract DNA, RNA, and protein from the same tissue in a single step, and it has the advantage that it extracts all RNA fragments, including lncRNAs. Furthermore, electric-powered homogenisation leads to better and finer disintegration of the underlying biomaterial, especially the vascular tissue, which is tough and thus hard to shred using manual tools.

Preserving high-quality RNA is a pivotal issue in achieving reliable data, particularly for new-generation omics analyses. Lu et al. [22] analysed the impact of RNA degradation on the next-generation sequencing using human cells with different degrees of RNA degradation (RINs ranging from 9.8 to 2.5). The authors showed that only slight changes in RNA degradation might significantly affect the expression levels of mRNAs as well as lncRNAs. However, the degradation process was global and random in most genes, and, interestingly, the pathway enrichment analysis was not affected. Consequently, even if the RNA quality is low, using proper controls with a similar degree of RNA disintegration might still provide consistent data. Generally, the RIN in a human tissue samples lies between 6.0 and 8.0, depending on the organ tissue [23]. Our results obtained from non-diseased human muscle samples confirmed these values (7.5 ± 0.9). In contrast, and as expected, the RINs of the diseased vascular tissue samples (AAA and PAD) were significantly lower (3.0 ± 0.6 and 3.1 ± 0.5, respectively). Because all tissue biomaterial collected in our SVB is frozen within 5 min following surgical excision, the low RINs are due to atherosclerotic changes and other pathologies within the diseased vascular tissues. In contrast to other human diseases, vascular disorders lead to RNA degradation through various accompanying processes such as cellular apoptosis, oxidative stress, inflammation, and calcification [11,12,13]. Calcified tissue samples from PAD patients are a particular challenge due to the low number of cells, the low amount of RNA, and the low quality of the extracted RNA. To date, few studies have focused on RNA integrity in tissue from patients with CVDs. Martinet et al. analysed 20 carotid plaques and found high heterogeneity in the degradation of the ribosomal genes *18S* and *28S*, depending on the extent of atherosclerosis [11]. Furthermore, the authors demonstrated the strong effect of oxidative damage on the quality of RNA. Our data were also in line with those of Tutino et al. [24], who analysed over 70 samples from patients with ischemic stroke and found an average RIN of 3.1.

Interestingly, the RNA quality in our healthy aortas obtained after kidney transplantation was significantly lower than expected and was comparable to that found in the diseased vascular tissues. The reason for the diminished RNA integrity was obviously the cold ischemia and the time span between tissue excision from the donor and organ transplantation into the recipient, which in our case takes approximately 14 to 16 h. Nonetheless, our histological characterisation of the healthy aortic tissue samples showed intact arterial walls without any signs of atherosclerotic changes. Even if the donor kidney, which contains renal artery tissue and a part of the abdominal aorta, is preserved in a specific protective solution, e.g., IGL-1 [25], cold ischemia will still have an effect. Oxygen deprivation results in metabolic substrate starvation, which may lead to cellular apoptosis, mitochondrial dysfunction, and thus to increased oxidative stress [26]. Such processes may cause RNA degradation in the remaining part of the aorta. Smooth muscle cells are particularly susceptible to apoptosis [27,28]. On the other hand, due to the partial RNA degradation of the healthy aortas, which we used as controls in our experimental approaches, these samples seem even more suitable for comparison with the diseased vascular tissues (AAA, PAD, and others). Because RNA degradation is random and global [22], affecting the RNA as a whole, including non-coding RNAs, the differences in RNA quality (RIN) between the study groups should not exceed the magnitude of 2.0 (according to the recommendation of the Functional Genomic Center Zurich, ETH Zurich, Switzerland).

Regarding the maintenance of RNA quality over time, the RNAlater solution showed the best protective properties, as expected. The RIN values were stable for up to 7 days at 4 °C. Similar results were observed for DV200. However, although the RNAlater solution seems to be the best solution for storing tissue samples for transcriptomics analyses, it can be difficult to use in combination with other biological and histological approaches, and the resulting RNA quality is often hampered. Furthermore, as soon as the tissue is transferred into the RNAlater solution, no living cells can be isolated. Therefore, we also tested the properties of our cryoprotective solution (CpS). The CpS contains various components which are intended to maintain cellular viability [16,17,18,19]. Furthermore, the CpS enables the freezing and thawing of the tissue without losing its integrity, allowing the isolation of primary vascular cells. Our CpS showed high protective properties against RNA degradation for up to 3 days. The lack of RNA stability over time was demonstrated, particularly in PBS. For the non-diseased muscle tissue, the first signs of RNA disintegration were observed after only 8 h. In contrast, in the healthy aorta samples (AAA, and PAD), the RINs were stable for up to 2–3 days. It seems that the highly sensitive and unstable RNAs in diseased vascular tissue degrade quickly, and the remaining RNAs are generally stable. Similar results were achieved for DV200. However, the DV200 index seemed to be more robust as a measure of RNA integrity than the RIN, particularly in the diseased vascular tissue. Interestingly, different results were achieved when the tissue samples were incubated at room temperature. Under these conditions, the RNA degradation proceeded much faster. Consequently, if it is not possible to freeze the tissue of interest immediately after surgical intervention, the samples should be kept in an appropriate protective solution at a temperature of least at 4 °C.

Regarding the analysis of the expressions of various genes included in our study, the housekeeping genes were mainly independent of the corresponding RINs. For both genes, *ACTB* and *GAPDH*, their expression was unaffected for up to 2–3 days, regardless of the protective solutions in which the tissue samples were stored at 4 °C. Furthermore, *ACTB* mRNA seemed to be more stable than that of *GAPDH*. It is worth noting that the expression level strongly depended on the selected primers, the size of the amplicon, and the cDNA area, where the primers bind [29,30]. Our own comparative experiments showed up to 1000-fold differences in the expression levels between amplicons selected near the 5’- or the 3’-end of the corresponding cDNA sequence. Consequently, because mRNA degradation generally starts at the 3’-end, we recommend using PCR amplicons < 130 bp and primer pairs near the 5’-end of the mRNA, preferably spanning an intron region, to detect potential DNA contamination. Concerning the expression of the ribosomal genes, their stability was comparable with that of the housekeeping genes. However, *18S* seemed to be less stable than *28S*. This observation is in line with those of Julian et al. [31], who compared various potential housekeeping genes for quantitative PCR analysis under hypoxic conditions and observed that *18S* was the least stable gene among those tested. In contrast, *ACTB* and *GAPDH* were stable and thus also suitable as reference genes in aged and diseased ischemic tissue [32,33]. Furthermore, Vazquez-Blomquist [34] investigated *18S* and *28S* gene stability in comparison to that of *ACTB* and *GAPDH* in glioblastoma-derived cells. Both ribosomal genes showed lower stability than the housekeeping genes. Thus, even if ribosomal genes are stable and can be used for the calculation of RNA quality (RIN, Agilent), they may not be suitable for diseased vascular tissue in which the RNA is already degraded at the time of extraction.

Our experimental setup demonstrated that lncRNAs were the least stable genes. Depending on the heterogeneity of the point zero expression, a significant reduction in the expression of the lncRNAs *MALAT1* and *H19* was observed after just 2 days’ incubation at 4 °C. *MALAT1* in particular seemed to degrade rapidly. Furthermore, in contrast to the other tested genes, whose expression levels declined slowly with about a two-fold reduction after 2–3 days, the expression level of the lncRNAs dropped rapidly, and more than ten-fold. Again, the highest gene expression stability was observed in the RNAlater solution, and good results were also achieved using the CpS. The expression of *MALAT1* and *H19* is significantly increased in atherosclerotic lesions and aneurysm tissues [7,20]. *MALAT1* is an important lncRNA that regulates the proliferation of ECs and VSMCs and promotes autophagy [21,35]. *H19* plays a crucial role in metabolic disorders by suppressing lipid metabolism and increasing their accumulation, contributing to the progression of atherosclerosis [36]. *H19* is also involved in inflammatory responses involving NF-kB, TNF-α, IL-1β, and IL-6. Generally, lncRNAs, including *MALAT1* and *H19*, are involved in many epigenetic regulatory processes, affecting their activity and time of action [37]. In addition, lncRNAs have a plethora of different functional responsibilities involving distinct biological mechanisms, including regulating chromatin structure, binding and affecting gene transcription, sponging miRNAs, and interacting with proteins, transcription factors, or other RNA molecules [38]. All of these circumstances can significantly affect RNA stability, particularly in diseased vascular tissue. Furthermore, even if the lncRNAs tested in our study were the least stable, they were detected in every tissue sample analysed. Consequently, under appropriate conditions, non-coding RNAs might also be suitable as reference genes, particularly in exploring the expressions of other lncRNAs.

The correlation analysis confirmed the results of the expression experiments. RNAlater demonstrated the highest protective capability against RNA degradation over time. The cryoprotective solution (CpS) used to preserve tissue samples collected in our SVB also showed a high protective capacity for RNA. Furthermore, the DV200 index demonstrated higher robustness and was not as strongly affected by time as the RIN. Focusing on RNA from non-diseased muscle tissue in PBS without any special protective properties, the expression of all the genes tested in our study significantly correlated with the RINs, as expected. These data show the importance of the RIN as a measure of RNA quality for reliable expression analyses, including transcriptomics and single-cell RNA sequencing. Interestingly, however, in the atherosclerotic and aneurysmal tissue samples, only the expression levels of lncRNAs were related to the RIN. These data suggest that when the RIN is low, as it often is in diseased vascular tissue, the RIN might not be the best indicator of whether the tissue sample is suitable for further expression analyses. In such cases, we would recommend using the DV200 index, which represents global RNA degradation (not only of the ribosomal genes *18S* and *28S*) and can more reliably aid decisions concerning further analyses.

## 5. Conclusions

Diseased vascular samples with extended atherosclerotic pathologies represent a particular challenge due to their low RNA quality. Our results demonstrated that RNA isolation with TRIZOL using homogeniser leads to the highest RNA concentration and quality. The advantages of TRIZOL are that it extracts all RNA fragments, including non-coding RNAs, it is cost-effective, and it is easy to handle. Our data showed that even if the specimens were appropriately handled (i.e., frozen within 5 min of surgical intervention), the quality of the RNA (RIN) was rather low (ranging between 2 and 4). We have assumed that in cases in which other research groups have found higher RIN values, this was probably because their vascular tissue samples were not as heavily affected by atherosclerotic and other cardiovascular pathologies. Regarding the protective solutions tested in our study, a cryoprotective solution, as suggested in the current work, might be a better option than RNAlater. Furthermore, because the RNA degradation in the atherosclerotic tissue proceeds randomly and globally, we recommend using proper corresponding controls with RINs in a comparable range. Otherwise, the transcriptomics data might convey differences in gene expression based on differences in RNA degradation rather than changes at the expression level of the corresponding genes. Concerning the expression analyses, the lncRNAs tested in our study were the least stable in our experimental approach. Finally, in the atherosclerotic samples, both ribosomal genes *18S* and *28S* were already degraded. Therefore, we believe that in diseased vascular tissue, DV200, which represents the quantity of RNA fragments longer than 200 bp out of the total RNA, is more suitable as a decision-making aid to help choose samples for next-generation sequencing approaches. Illumina recommends a DV200 > 30% [39]. Based on our own experience, we suggest selecting samples, whenever possible, with a DV200 > 50%.

## Figures and Tables

**Table 1:** Correlation analysis.

	**Muscle**								
		**RIN**	**DV200**	**ACTB**	**GAPDH**	**RS18**	**RS28**	**MALAT1**	**H19**
**CpS**	**Time**	−0.706 **	n.s.	n.s.	−0.564 **	n.s.	−0.545 **	−0.632 **	−0.745 ***
	**RIN**		0.601 *	n.s.	n.s.	0.715 **	0.794 ***	0.794 ***	0.683 **
	**DV200**			n.s.	n.s.	n.s.	n.s.	n.s.	n.s.
**PBS**	**Time**	−0.922 ***	n.s.	−0.545 *	−0.652 **	−0.521 *	−0.912 ***	−0.681 **	−0.692 **
	**RIN**		n.s.	0.645 **	0.583 *	0.508 *	0.878 ***	0.885 ***	0.686 **
	**DV200**			n.s.	n.s.	n.s.	n.s.	n.s.	n.s.
**RNALater**	**Time**	−0.467 *	n.s.	n.s.	n.s.	n.s.	−0.545 *	n.s.	n.s.
	**RIN**		n.s.	n.s.	n.s.	n.s.	n.s.	n.s.	n.s.
	**DV200**			0.543 *	n.s.	n.s.	n.s.	n.s.	n.s.
	**Healthy aorta**								
		**RIN**	**DV200**	**ACTB**	**GAPDH**	**RS18**	**RS28**	**MALAT1**	**H19**
**CpS**	**Time**	n.s.	n.s.	n.s.	n.s.	n.s.	−0.520 *	−0.707 ***	−0.647 **
	**RIN**		n.s.	n.s.	n.s.	n.s.	n.s.	n.s.	n.s.
	**DV200**			n.s.	n.s.	0.510 *	0.713 **	n.s.	n.s.
**PBS**	**Time**	−0.685 **	−0.873 ***	−0.615 **	−0.771 ***	−0.623 **	−0.529 *	−0.737 ***	−0.561 *
	**RIN**		0.743 **	n.s.	0.533 *	0.517 *	n.s.	0.490 *	n.s.
	**DV200**			0.523 *	0.785 ***	0.629 *	0.553 *	0.763 ***	0.577 *
**RNALater**	**Time**	n.s.	n.s.	n.s.	n.s.	n.s.	n.s.	n.s.	n.s.
	**RIN**		n.s.	n.s.	n.s.	n.s.	n.s.	n.s.	n.s.
	**DV200**			n.s.	n.s.	n.s.	n.s.	n.s.	n.s.
	**AAA**								
		**RIN**	**DV200**	**ACTB**	**GAPDH**	**RS18**	**RS28**	**MALAT1**	**H19**
**CpS**	**Time**	−0.650 **	n.s.	−0.540 *	−0.705 **	−0.720 ***	−0.557 *	−0.764 ***	−0.591 **
	**RIN**		0.541 *	n.s.	0.674 **	0.621 **	n.s.	0.497 *	0.520 *
	**DV200**			n.s.	n.s.	n.s.	0.676 **	0.558 *	n.s.
**PBS**	**Time**	−0.542 *	n.s.	−0.506 *	−0.863 ***	−0.779 ***	−0.644 **	−0.914 ***	−0.669 **
	**RIN**		n.s.	n.s.	n.s.	0.503 *	n.s.	0.569 *	0.458 *
	**DV200**			n.s.	n.s.	n.s.	n.s.	n.s.	n.s.
**RNALater**	**Time**	n.s.	n.s.	n.s.	n.s.	n.s.	n.s.	n.s.	n.s.
	**RIN**		n.s.	n.s.	n.s.	n.s.	n.s.	n.s.	0.459 *
	**DV200**			0.608 *	n.s.	0.625 *	n.s.	n.s.	n.s.
	**PAD**								
		**RIN**	**DV200**	**ACTB**	**GAPDH**	**RS18**	**RS28**	**MALAT1**	**H19**
**CpS**	**Time**	−0.604 **	−0.505 *	−0.644 **	−0.739 ***	−0.798 ***	−0.578 **	−0.771 ***	n.s.
	**RIN**		n.s.	0.518 *	n.s.	n.s.	n.s.	n.s.	0.584 **
	**DV200**			n.s.	n.s.	n.s.	n.s.	n.s.	n.s.
**PBS**	**Time**	−0.550 *	−0.640 *	−0.776 ***	−0.831 ***	−0.836 ***	−0.703 ***	−0.815 ***	−0.466 *
	**RIN**		n.s.	0.465 *	n.s.	n.s.	n.s.	0.486 *	n.s.
	**DV200**			0.732 **	n.s.	n.s.	n.s.	0.764 **	n.s.
**RNALater**	**Time**	n.s.	n.s.	n.s.	n.s.	n.s.	−0.481 *	−0.751 **	n.s.
	**RIN**		n.s.	n.s.	n.s.	n.s.	n.s.	0.454 *	n.s.
	**DV200**			n.s.	n.s.	n.s.	n.s.	n.s.	0.536 *

* *p* < 0.05, ** *p* < 0.01, *** *p* < 0.001. n.s.: not significant.

## Data Availability

Not applicable.

## References

[1] Hentze J.L., Kringelbach T.M., Novotny G.W., Hamid B.H., Ravn V., Christensen I.J., Hogdall C., Hogdall E. (2019). Optimized Biobanking Procedures for Preservation of RNA in Tissue: Comparison of Snap-Freezing and RNAlater-Fixation Methods. Biopreserv. Biobank..

[2] Pelisek J., Hegenloh R., Bauer S., Metschl S., Pauli J., Glukha N., Busch A., Reutersberg B., Kallmayer M., Trenner M. (2019). Biobanking: Objectives, Requirements, and Future Challenges-Experiences from the Munich Vascular Biobank. J. Clin. Med..

[3] Vaduganathan M., Mensah G.A., Turco J.V., Fuster V., Roth G.A. (2022). The Global Burden of Cardiovascular Diseases and Risk: A Compass for Future Health. J. Am. Coll. Cardiol..

[4] Keegan A., Hicks C.W. (2022). Surgical Decision-Making and Outcomes in Open Versus Endovascular Repair for Various Vascular Diseases. Anesthesiol. Clin..

[5] Anagnostakos J., Lal B.K. (2021). Abdominal aortic aneurysms. Prog. Cardiovasc. Dis..

[6] Klaus V., Tanios-Schmies F., Reeps C., Trenner M., Matevossian E., Eckstein H.H., Pelisek J. (2017). Association of Matrix Metalloproteinase Levels with Collagen Degradation in the Context of Abdominal Aortic Aneurysm. Eur. J. Vasc. Endovasc. Surg..

[7] Li D.Y., Busch A., Jin H., Chernogubova E., Pelisek J., Karlsson J., Sennblad B., Liu S., Lao S., Hofmann P. (2018). H19 Induces Abdominal Aortic Aneurysm Development and Progression. Circulation.

[8] Pelisek J., Eckstein H.H., Zernecke A. (2012). Pathophysiological mechanisms of carotid plaque vulnerability: Impact on ischemic stroke. Arch. Immunol. Ther. Exp..

[9] Reeps C., Kehl S., Tanios F., Biehler J., Pelisek J., Wall W.A., Eckstein H.H., Gee M.W. (2014). Biomechanics and gene expression in abdominal aortic aneurysm. J. Vasc. Surg..

[10] Ahmed S., Shaffer A., Geddes T., Studzinski D., Mitton K., Pruetz B., Long G., Shanley C. (2015). Evaluation of optimal RNA extraction method from human carotid atherosclerotic plaque. Cardiovasc. Pathol..

[11] Martinet W., de Meyer G.R., Herman A.G., Kockx M.M. (2004). Reactive oxygen species induce RNA damage in human atherosclerosis. Eur. J. Clin. Investig..

[12] Martinet W., De Meyer G.R., Herman A.G., Kockx M.M. (2005). RNA damage in human atherosclerosis: Pathophysiological significance and implications for gene expression studies. RNA Biol..

[13] Gallego Romero I., Pai A.A., Tung J., Gilad Y. (2014). RNA-seq: Impact of RNA degradation on transcript quantification. BMC Biol..

[14] Matsubara T., Soh J., Morita M., Uwabo T., Tomida S., Fujiwara T., Kanazawa S., Toyooka S., Hirasawa A. (2020). DV200 Index for Assessing RNA Integrity in Next-Generation Sequencing. Biomed. Res. Int..

[15] Towler B.P., Newbury S.F. (2018). Regulation of cytoplasmic RNA stability: Lessons from Drosophila. Wiley Interdiscip. Rev. RNA.

[16] Bakhach J. (2009). The cryopreservation of composite tissues: Principles and recent advancement on cryopreservation of different type of tissues. Organogenesis.

[17] Elliott G.D., Wang S., Fuller B.J. (2017). Cryoprotectants: A review of the actions and applications of cryoprotective solutes that modulate cell recovery from ultra-low temperatures. Cryobiology.

[18] Muller-Schweinitzer E. (2009). Cryopreservation of vascular tissues. Organogenesis.

[19] Taylor M.J., Weegman B.P., Baicu S.C., Giwa S.E. (2019). New Approaches to Cryopreservation of Cells, Tissues, and Organs. Transfus. Med. Hemother..

[20] Cao M., Luo H., Li D., Wang S., Xuan L., Sun L. (2022). Research advances on circulating long noncoding RNAs as biomarkers of cardiovascular diseases. Int. J. Cardiol..

[21] Josefs T., Boon R.A. (2020). The Long Non-coding Road to Atherosclerosis. Curr. Atheroscler. Rep..

[22] Lu W., Zhou Q., Chen Y. (2022). Impact of RNA degradation on next-generation sequencing transcriptome data. Genomics.

[23] Walker D.G., Whetzel A.M., Serrano G., Sue L.I., Lue L.F., Beach T.G. (2016). Characterization of RNA isolated from eighteen different human tissues: Results from a rapid human autopsy program. Cell Tissue Bank..

[24] Tutino V.M., Fricano S., Frauens K., Patel T.R., Monteiro A., Rai H.H., Waqas M., Chaves L., Poppenberg K.E., Siddiqui A.H. (2021). Isolation of RNA from Acute Ischemic Stroke Clots Retrieved by Mechanical Thrombectomy. Genes.

[25] Habran M., De Beule J., Jochmans I. (2020). IGL-1 preservation solution in kidney and pancreas transplantation: A systematic review. PLoS ONE.

[26] Kosieradzki M., Rowinski W. (2008). Ischemia/reperfusion injury in kidney transplantation: Mechanisms and prevention. Transplant. Proc..

[27] Kim S.M., Huh J.W., Kim E.Y., Shin M.K., Park J.E., Kim S.W., Lee W., Choi B., Chang E.J. (2019). Endothelial dysfunction induces atherosclerosis: Increased aggrecan expression promotes apoptosis in vascular smooth muscle cells. BMB Rep..

[28] Solomon C.U., McVey D.G., Andreadi C., Gong P., Turner L., Stanczyk P.J., Khemiri S., Chamberlain J.C., Yang W., Webb T.R. (2022). Effects of Coronary Artery Disease-Associated Variants on Vascular Smooth Muscle Cells. Circulation.

[29] Bustin S., Huggett J. (2017). qPCR primer design revisited. Biomol. Detect. Quantif..

[30] Bustin S., Nolan T. (2017). Talking the talk, but not walking the walk: RT-qPCR as a paradigm for the lack of reproducibility in molecular research. Eur. J. Clin. Investig..

[31] Julian G.S., de Oliveira R.W., Perry J.C., Tufik S., Chagas J.R. (2014). Validation of housekeeping genes in the brains of rats submitted to chronic intermittent hypoxia, a sleep apnea model. PLoS ONE.

[32] Ju W., Smith A.O., Sun T., Zhao P., Jiang Y., Liu L., Zhang T., Qi K., Qiao J., Xu K. (2018). Validation of Housekeeping Genes as Reference for Reverse-Transcription-qPCR Analysis in Busulfan-Injured Microvascular Endothelial Cells. Biomed. Res. Int..

[33] Yang M., Yan J., Wu A., Zhao W., Qin J., Pogwizd S.M., Wu X., Yuan S., Ai X. (2021). Alterations of housekeeping proteins in human aged and diseased hearts. Pflügers Arch.-Eur. J. Physiol..

[34] Vazquez-Blomquist D., Fernandez J.R., Miranda J., Bello C., Silva J.A., Estrada R.C., Novoa L.I., Palenzuela D., Bello I. (2012). Selection of reference genes for use in quantitative reverse transcription PCR assays when using interferons in U87MG. Mol. Biol. Rep..

[35] Busscher D., Boon R.A., Juni R.P. (2022). The multifaceted actions of the lncRNA H19 in cardiovascular biology and diseases. Clin. Sci..

[36] Shi X., Wei Y.T., Li H., Jiang T., Zheng X.L., Yin K., Zhao G.J. (2020). Long non-coding RNA H19 in atherosclerosis: What role?. Mol. Med..

[37] Skuratovskaia D., Vulf M., Komar A., Kirienkova E., Litvinova L. (2019). Promising Directions in Atherosclerosis Treatment Based on Epigenetic Regulation Using MicroRNAs and Long Noncoding RNAs. Biomolecules.

[38] Schober A., Maleki S.S., Nazari-Jahantigh M. (2022). Regulatory Non-coding RNAs in Atherosclerosis. Prevention and Treatment of Atherosclerosis: Improving State-of-the-Art Management and Search for Novel Targets. Handb. Exp. Pharmacol..

[39] Illumina Evaluating RNA Quality from FFPE Samples. https://emea.illumina.com/content/dam/illumina-marketing/documents/products/technotes/evaluating-rna-quality-from-ffpe-samples-technical-note-470-2014-001.pdf.

